# Effect of Steviol Glycosides on Human Health with Emphasis on Type 2 Diabetic Biomarkers: A Systematic Review and Meta-Analysis of Randomized Controlled Trials

**DOI:** 10.3390/nu11091965

**Published:** 2019-08-21

**Authors:** Camilla Christine Bundgaard Anker, Shamaila Rafiq, Per Bendix Jeppesen

**Affiliations:** Department of Clinical Medicine, Aarhus University, Aarhus University Hospital, Palle Juul-Jensens Boulevard 165, 8200 Aarhus N, Denmark

**Keywords:** steviol glycosides, type 2 diabetes, fasting blood glucose, lipids, BMI, blood pressure, HbA1c

## Abstract

The natural sweetener from *Stevia rebaudiana* Bertoni, steviol glycoside (SG), has been proposed to exhibit a range of antidiabetic properties. The objective of this systematic review was to critically evaluate evidence for the effectiveness of SGs on human health, particularly type 2 diabetic (T2D) biomarkers, collecting data from randomized controlled trials (RCTs). Electronic searches were performed in PubMed and EMBASE and the bibliography of retrieved full-texts was hand searched. Using the Cochrane criteria, the reporting quality of included studies was assessed. Seven studies, nine RCTs, including a total of 462 participants were included. A meta-analysis was performed to assess the effect of SGs on following outcomes: BMI, blood pressure (BP), fasting blood glucose (FBG), lipids, and glycated hemoglobin (HbA1c). The meta-analysis revealed an overall significant reduction in systolic BP in favour of SGs between SG and placebo, mean difference (MD): −6.32 mm Hg (−7.69 to 0.46). The overall effect of BMI, diastolic BP, FBG, total cholesterol, and high-density lipoprotein cholesterol (HDL-C) was a non-significant reduction in favour of SGs, and a non-significant increase in low-density lipoprotein cholesterol and triglyceride, while no significant effect of HbA1c was found. Heterogeneity was significant for several analyses. More studies investigating the effect of SGs on human health, particularly T2D biomarkers, are warranted.

## 1. Introduction

Diabetes mellitus has been ranked as the sixth leading cause of disability [1]. The International Diabetes Federation (IDF) estimated a total of more than 425 million diabetics in the age range 20–79 worldwide in 2017, type 2 diabetes mellitus (T2DM) accounting for more than 90% of the overall cases. According to IDF, the number of T2DM incidences is expected to further increase to around 629 million by year 2045, making this metabolic disease a continuously increasing problem worldwide [2,3].

T2DM is a metabolic and multifactorial condition in that both genetic, epigenetic, and environmental factors, including diet and physical activity, contribute to the development of the disease [4]. In most cases, insulin resistance is present and precedes the development of T2DM by increasing the requirements for insulin, leading to insulin insufficiency in individuals whose β-cells have limited secretory reserve and is most often related to obesity, ageing, and physical inactivity [5,6]. Indeed, it is suggested that the case of insulin resistance as seen in many T2DM patients is the result of an increase in visceral adiposity [5,7]. As a consequence of the insulin deficiency, there is a reduced insulin-mediated glucose uptake from skeletal muscle and other peripheral target tissues and an increased glucose production from the liver, as a result of increased glucagon secretion from the α-cells, and free fatty acid mobilization from adipose tissue. Initially, this will cause postprandial hyperglycemia, eventually resulting in fasting hyperglycemia [7,8].

Left untreated, T2DM might eventually lead to secondary complications such as microvascular and macrovascular complication and cause morbidity and mortality [5,9].

Several chemical drugs have been developed in order to manage and treat T2DM of which metformin is the first drug of choice. Metformin affects insulin sensitivity to increase glucose uptake in the liver. Previously, glitazones have also been administered to type 2 diabetic patients to increase insulin sensitivity in muscles. However, due to severe side effects, these agents have almost been removed from the market. Furthermore, SGLT-2 and DPP-4 inhibitors have been introduced as antidiabetic agents. SGLT-2 inhibitors reduce the blood glucose by inhibiting SGLT-2 and thereby increasing the excretion of glucose in the urine. DPP-4 inhibitors stimulate the insulin secretion while inhibiting degradation of the insulinotropic hormone GLP-1, resulting in a blood glucose reduction. Some agents exhibit insulin-resistance-reducing effects and might reduce the requirement for insulin secretagogues such as sulfonylureas and meglitinides. However, none of the existing drugs have a completely healing effect, and most of the currently available chemical drugs are costly and cause side effects [6,10]. 

In the search of alternative treatments, natural products extracted from a number of herbal plants have been found to exhibit hypoglycemic activity [11,12,13]. The natural constituents in the leaves of the herb *Stevia rebaudiana* Bertoni have been closely investigated in a range of in vitro, in vivo, and human studies, some demonstrating the possession of Stevioside and other SGs of some antidiabetic capabilities in both humans [14,15,16,17,18,19,20,21,22,23,24,25] and rodents [26,27,28,29,30,31,32]. 

### 1.1. Stevia Rebaudiana Bertoni

The perennial shrub *Stevia rebaudiana* Bertoni belongs to the *Asteraceae* family native to South America, in particular Paraguay and Brazil. For decades, indigenous people have used extracts from the leaves of this sweet herb as sweetener to several foods and beverages and in medicines, e.g., for treatment of diabetes [33]. High-purity steviol glycoside extracts (≥95%) have been approved for use as food sweetener in several countries and regions, including the European Union and the United States. The sweet taste is a result of the presence of the natural constituents of the plant known as steviol glycosides (SGs) [34,35]. 

**Figure 1:**
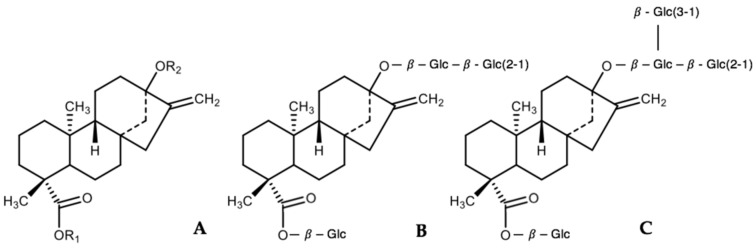
Backbone structure of SGs (**A**), Stevioside (**B**), and Reb A (**C**).

The SGs derived from the plant are four-ring diterpenes composed of an aglycone backbone called steviol to which various numbers and types of sugars are attached (Figure 1A). Presently, >40 SGs have been identified, Stevioside (4–13% wt:wt, Figure 1B) and Rebaudioside A (Reb A—2–4% wt:wt, Figure 1C) being the most abundant glycosides in the leaves of *S. rebaudiana*. Stevioside and Reb A are both non-caloric compounds ∼200–300 times sweeter than 0.4 M sucrose and are chemically very similar, differing only by one additional glucose moiety on Reb A. In general, SGs differ only in the number and type of monosaccharides attached to the aglycone [36]. 

#### 1.1.1. Metabolism of Steviol Glycosides

Besides sharing similar chemical structure, SGs also experience the same metabolic fate. Due to the lack of capability of the enzymes and acid present in the upper gastrointestinal tract to digest SGs, SGs remains undigested and enter the colon where the microbiota is responsible for hydrolysis and degradation processes. The glycosidic linkages are cleaved and the sugar moieties removed, leaving behind the aglycone backbone, steviol. A large amount of steviol is quickly absorbed and transported to the liver where it is conjugated to glucuronic acid to form steviol glucuronide (SVG), the rest is excreted in the feces. Finally, the end product, SVG, is excreted via urine in humans and via bile in rats. Besides minor differences in the metabolism rate accounted for by the number of sugar moieties present, SGs are hydrolyzed to steviol at generally similar rates, regardless of the type or numbers of sugar moieties [37,38,39,40]. 

#### 1.1.2. Effect of Steviol Glycosides on Health and Diabetes Biomarkers

A number of studies in both human and rodents have been conducted to investigate the effects of SGs on diabetes biomarkers such as blood pressure (BP), fasting blood glucose, and insulin etc., and it has been reported that SGs exhibit some antidiabetic capabilities. 

### 1.2. Effect on Fasting Blood Glucose and Insulin

An early study by Curi et al. [41] showed a correlation between ingestion of extracts of *S. rebaudiana* Bertoni and suppression of plasma glucose and an increase in glucose tolerance in normal adult humans.

Subsequently, long-term human trials have been conducted, aiming to investigate the effect of 200–1500 mg/day orally administered stevioside [15,16,17,19,42,43] and 500–1000 mg/day Reb A [22,23] in a time period ranging from 3 days to 2 years on glycated hemoglobin (HbA1c), fasting blood glucose, and insulin in healthy, type 1 and type 2 diabetic, low-normotensive, hypertensive, and hyperlipidemic subjects. Neither of the investigated outcomes were found to be significantly changed when comparing intervention groups with control groups. However, it should be noted that the protocols of the studies enrolling diabetic subjects differed with some continuing their antidiabetic medications [15,23] and some ending before initiation of the study [22,43]. 

In one of these studies including diabetic subjects continuing their hypoglycemic medications [15], oral administration of 750 mg/day Stevioside for 3 months did not affect the blood glucose and HbA1c levels in both type 2 diabetics and normotensive/low-normotensive subjects. Although the Stevioside treatment was found not to exert any hypoglycemic effect in the subjects, a significant increase in fasting blood glucose concentration was indeed detected in the placebo group compared with baseline in subjects with type 1 diabetes mellitus, whereas the subjects consuming Stevioside managed to maintain the fasting blood glucose concentration during the 3 months study period. 

Similar results were revealed in a study by Jeppesen et al. [43] where 55 type 2 diabetics ending their hypoglycemic medications at initiation of 1500 mg/day Stevioside treatment for 3 months. Both HbA1c and fasting blood glucose were found to be significantly increased for the placebo group (*p* < 0.01 and *p* < 0.007, respectively) during the 3 months, whereas for the Stevioside group, no change was found in the measures (*p* < 0.14 and *p* < 0.1, respectively). 

A non-significant reduction in both HbA1c and fasting blood glucose levels in type 2 diabetic subjects post treatment of 1000 mg/day Reb A for 16 weeks was reported by Maki et al. [22]. However, a slight rise in HbA1c levels was observed in both Reb A and placebo groups and no difference in HbA1c changes for Reb A compared with placebo was observed. Moreover, the fasting insulin increased significantly in the Reb A group compared with placebo. However, the changes from baseline to treatment were not different between groups.

A study examined the effect of 1 g/day Stevia leaf powder for 60 days on fasting blood glucose in type 2 diabetic subjects [44]. Here, the volunteers were treated with Stevia leaf powder in preference to high-purity SGs as seen in the protocols of the other studies. Ritu et al. reported significantly reduced fasting blood glucose levels of diabetic subjects of the intervention groups 60 days post initiation of treatment compared with prior to intervention [44]. However, these results should be interpreted with caution, since crude Stevia leaf powder contain a number of other components which might influence and affect the results. 

In a meta-analysis, Onakpoya and Heneghan [24] evaluated existing evidence for effectiveness on cardiovascular risk factors in adults, using data from RCTs. An evaluation of six of aforementioned studies showed a small but significant decrease in fasting blood glucose (−0.63 mmol/L, *p* < 0.00001). It is discussed whether this observation is of clinical relevance due to the limited extent of reduction [36].

According to Jeppesen et al. [43], one possible reason for these contrary findings compared to data from several animal studies is the enrollment of diabetic subjects who might have been at a late developmental stage of the disease, and consequently with limited β-cell function. 

Although no evidence indicates a direct hypoglycemic or insulinotropic effect of Stevia and its SGs in both healthy and diabetic human subjects, findings suggest that a potential of these to maintain a static diabetic state exists at levels above ADI, i.e., 0–4 mg steviol equivalents (SEs)/kg bw/day [36]. 

### 1.3. Effect on Energy Intake and Weight Control

From findings that Stevia might possess beneficial effects on blood glucose and insulin levels in humans [18,41], it has been suggested that Stevia also might have a role in food intake regulation. To date, only two studies have investigated the effects of Stevia on satiety and food/energy intake [14,25].

In a randomized crossover study, Anton et al. tested the effects of two preloads containing either Stevia (580 kcal), aspartame (580 kcal), or sucrose (986 kcal) consumed 20 min prior to ad libitum test meals two times daily for three days on food intake and satiety, among other factors, in both lean and obese individuals [14]. A significant reduction in food intake over the entire day (including preloads) was observed in the Stevia group compared to the sucrose group. However, when excluding the preload calories from the analyses, no significant difference in food intake was observed. In addition, the investigators did not find any differences in satiety levels between the different conditions at any time point, all indicating that the participants did not compensate by eating more at the meals at lunch or dinner.

In a second randomized cross-over study, Tey et al. examined the effects of Stevia (0.33 g; Reb A) compared to sucrose (65 g) in liquid form on total daily energy intake in 30 healthy, relatively lean males [25]. The study beverage was consumed 1 h prior to an ad libitum study lunch. Stevia was found not to reduce the total daily energy intake, with 73% compensation of the energy obtained from sucrose. Neither did Stevia affect the satiety ratings. In fact, Stevia was shown to significantly increase the desire to eat, hunger, and prospective consumption ratings 30 min post treatment and immediately before consumption of the ad libitum lunch compared with the sucrose treatment. Additionally, the included subjects reported lower fullness for the Stevia treatment compared with the control. 

When including findings from clinical trials investigating BMI and body weight as second outcomes, similar results are reported indicating no change in these measures post treatment of Stevia compared with placebo treatment [16,17,19,22]. 

### 1.4. Effect on Blood Pressure

In vivo studies have suggested the ability of intravenously administered Stevioside to cause a hypotensive effect [45,46]. However, it has been discussed whether the effects are the same after oral administration in humans, since the uptake of Stevioside has been shown to be extremely low as a consequence to its high molecular weight [42,47]. 

The effect of SGs on both systolic and diastolic blood pressure has been investigated in randomized clinical trials (RCTs) in both healthy (normotensive), hypertensive, and type 1 and type 2 diabetic adults. Geuns et al. [20] revealed that neither systolic blood pressure (SBP) or diastolic blood pressure (DBP) in healthy, normotensive volunteers were affected after oral administration of 750 mg/day Stevioside for 3 days. This in agreement with findings by Barriocanal et al. [15], demonstrating that oral administration of 750 mg/day Stevioside for 3 months does not exhibit any hypotensive effect in subjects with normal/low-normal BP levels. Likewise, the blood pressure of type 2 diabetic subjects was not affected by the administration of either 1000 mg/day Reb A for 16 weeks [22] or 750 mg/day [15] or 1500 mg/day [43] Stevioside for 3 months. However, for these studies, continuation of hypotensive medications among the hypertensive subjects during the study period was part of the protocol. 

The possible hypotensive effect of Stevioside was investigated in two randomized, double-blinded, placebo-controlled studies including hypertensive subjects not ending treatment with BP reducing medications before study initiation [16,19]. In a study enrolling 168 subjects with mild essential hypertension, 1500 mg/day orally administrated Stevioside for two years was shown to significantly reduce the SBP and DBP compared with baseline in the Stevioside group [19]. A significant reduction was also observed when comparing the Stevioside group and the placebo group. Furthermore, it was reported that the blood pressure started decreasing ∼1 week after initiation of the Stevioside administration [19]. This agrees with findings by Chan et al. [16], conducting a similar study investigating the effectiveness and tolerability of 750 mg/day Stevioside in hypertensive patients. From this study, the reduction in BP was registered 7 days after initiation of the active treatment, and the hypotensive effect of Stevioside reached statistical significance after 3 months when comparing the Stevioside and the placebo group. 

SGs were shown to cause a significant decrease in diastolic blood pressure (−2.24 mm Hg; *p* = 0.03) in a meta-analysis of seven RCTs [24]. However, non-significant results were obtained regarding SGs effect on systolic blood pressure, probably due to the observed significant heterogeneity between the included studies. As already stated, protocols of some of the included studies regarding continuation of blood pressure lowering and antidiabetic medications were not concordant, leading to inconsistency and incommensurability of the data presented in the studies.

### 1.5. Effect on Lipids

A few studies have included measures of lipids such as cholesterols, triacylglycerols (TAGs), and free fatty acids (FFAs) as part of the investigation of the effects of Stevia and SGs on human health and diabetic biomarkers [15,16,17,19,22,43,44]. 

Most of the existing literature indicate no effect of 200 mg/day–1500 mg/day Stevioside and 1000 mg/day Reb A on lipids in mild hypertensive, hypertensive, type 1 and type 2 diabetic, and healthy subjects when administered orally for 90 days to 2 years. 

In one study enrolling 49 hyperlipidemic volunteers [17], administration of 200 mg/day Stevioside did not exert any hypolipidemic effect. A decrease in blood levels of total cholesterol and LDL values during the treatment was detected in both Stevioside and placebo groups; however, no change in BMI was reported. 

When administering 1 g Stevia leaf powder for 60 days in type 2 diabetic subjects, the serum cholesterol, triglyceride, and very low-density lipoprotein-cholesterol (VLDL-C) levels were found to be significantly reduced [44].

### 1.6. Mechanism of Hypoglycemic, Insulinotropic, and Hypotensive Effects

To investigate the possible capability of SGs to induce a maintaining effect of glucose homeostasis, in vitro and animal studies have been conducted. SGs are suggested to exert hypoglycemic, insulinotropic, and glucagonostatic effects through direct or indirect action on mechanisms involving insulin secretion, signaling, and release; glucagon secretion, and release; regulation of key genes; and glucose absorption. It is obvious that the substitution of sucrose or other carbohydrates with non-caloric or non-nutritive sweeteners, such as SGs, will result in a lowering of the blood glucose concentrations and thereby a stable state of glycemia [48].

However, from studies observing diabetic Goto–Kakizaki (GK) rats [26] and streptozotocin (STZ)- or fructose-induced diabetic male Wistar rats [29,31], both intravenously and orally administered Stevioside was shown to exert antihyperglycemic, insulinotropic, and glucagonostatic actions, these effects being more prominent after a glucose load. In addition, in vitro insulin action on skeletal muscle glucose transport in both lean and obese Zucker rats has been shown to be improved by 0.01–0.1 mM Stevioside [49].

Jeppesen et al. [26] investigated the role of Stevioside in the management of glycemia in both the nonobese animal model of type 2 diabetes, GK rats, and normal Wistar rats. During an intravenous glucose tolerance test (GTT) with and without 0.2 g/kg Stevioside in anesthetized GK rats, Stevioside induced a significant reduction in blood glucose (*p* < 0.05), an increase in insulin secretion (*p* < 0.05), and a reduction in glucagon levels. No effect was observed in normal Wistar rats after administration of Stevioside. Jeppesen et al. [32] further showed that long-term administration of Stevioside at a dose of 0.025 g/kg/day resulted in suppression of plasma glucose after intra-arterial injection of a bolus of 2 g/kg glucose in GK rats (incremental AUC, *p* < 0.05). In addition, a higher first phase insulin response (incremental AUC, *p* < 0.05) was evident from the Stevioside-fed animals compared with the control group, causing a concomitant suppression of glucagon (incremental AUC, *p* < 0.05). However, no effect of the long-term administration on fasting blood glucose, insulin, and glucagon was reported. Similar observations were reported in a previous study by Jeppesen et al. [27], demonstrating the capability of Stevioside and the aglycone to potentiate insulin secretion from isolated mouse islets in a dose- and glucose-dependent manner. 

Fructose-rich chow fed to normal Wistar rats induced a diabetic state in the animals which oral administration of 0.5 mg/kg to 5.0 mg/kg Stevioside seemed to overcome [31]. A single oral administration of 0.5 mg/kg Stevioside for 90 min decreased plasma glucose concentrations and lowered the glucose-insulin index (a measure of insulin resistance) during an intraperitoneal glucose tolerance test in the fructose-rich chow-fed rats. Repeated oral administration of 5.0 mg/kg Stevioside resulted in a significant decrease in plasma glucose (*p* < 0.01) and a reduction in fasting plasma insulin in rats fed fructose-rich chow for four weeks. Furthermore, Stevioside improved insulin resistance induced by food, and delayed the induction of insulin resistance in the high-fructose chow fed animals together with a reported ability of increasing the insulin response, and thereby insulin sensitivity, in STZ-diabetic rats. Noteworthy, Stevioside (10 days after treatment) was found to improve insulin sensitivity in these rats more rapidly compared to metformin (15 days after treatment). Similar findings were reported by Chen et al. [29]. Stevioside administered via gastrogavage lowered the blood glucose levels in normal Wistar rats (in a dose dependent manner, 0.5 mg/kg to 5.0 mg/kg) and lowered high glucose levels in STZ-induced diabetic rats, and in fructose-induced diabetic rats. For both STZ-induced diabetic rats and fructose-induced diabetic rats, Stevioside treatment by gastrogavage lowered the blood glucose levels after just one day compared to rats in the control group. 

In a nine-week intervention, Nordentoft et al. demonstrated that the more bioavailable Stevioside derivative, isosteviol (ISV), was capable of decreasing the glucose-insulin index, i.e., reducing insulin resistance, and preventing development of a severe state of diabetes when fed to genetically obese diabetic KKay mice at a dose of 20 mg/kg/day [30]. In addition, ISV was found to upregulate the gene expression of the *GLUT2* glucose transporter protein gene transcript, key insulin-regulating genes and insulin transcription factors in pancreatic islets from KKAy control and ISV-treated mice, suggesting an enhanced glucose sensitivity as well as improved insulin expression and secretion in β-cells. 

In vitro studies using the clonal β-cell line, INS-1, and isolated Noval Medical Research Institute (NMRI) mice islets, have demonstrated increased glucose-stimulated insulin secretion (GSIS) when chronically exposed to 1 µmol/L to Stevioside or steviol [32] or ISV at 10^−10^ to 10^−6^ mol/L [30]. Jeppesen et al. presumed these changes to be partially caused by the concomitant capability of Stevioside and steviol to induce proinsulin, insulin, and a range of genes involved in glycolysis together with an upregulation in gene expression of glucose responsive genes and improvement of nutrient-sensing mechanisms in the INS-1 cells treated with Stevioside or the aglycone. Chen et al. [29] suggested the hypoglycemic effects of Stevioside to be caused by the effect of Stevioside on gluconeogenesis. In fact, it was shown that daily oral treatment with Stevioside decreased mRNA and protein levels of a rate-limiting enzyme for gluconeogenesis, PEPCK in diabetic rats. Blood glucose levels might be regulated and reduced in diabetic rats through a decreasing effect of Stevioside on the gene expression of PEPCK in the liver to slow down gluconeogenesis. Interestingly, aqueous extract from *S. rebaudiana* Bertoni has been shown to affect several mitochondrial functions [50]. An inhibitory effect of aqueous extract from *S. rebaudiana* Bertoni on the adenosine triphosphate (ATP) phosphorylation and nicotinamide adenine dinucleotide (NADH)-oxidase activity in rat liver mitochondria was reported. This contributed to an inhibition of adenosine diphosphate (ADP)/ATP exchange, resulting in increased glycolysis and decreased gluconeogenesis. 

In an additional study, Jeppesen et al. aimed to investigate the mechanism through which Stevioside and steviol (1 nmol/L to 1 mmol/L) exert an insulinotropic effect in INS-1 cells [27]. It was questioned whether the insulinotropic effects were mediated via the same mechanisms as the classic sulfonylureas, i.e., binding to receptor proteins on β-cells, blocking $K_{\mathrm{ATP}}^{+}$-channels and depolarizing the β-cell plasma membrane, inducing insulin release. Interestingly, neither of the diterpenes were found to possess a blocking capability of the plasma membrane $K_{\mathrm{ATP}}^{+}$-channels on β-cells, suggesting another mechanism to the insulinotropic effects than mediation via membrane depolarization as a consequence of closure of $K_{\mathrm{ATP}}^{+}$-channels in the β-cell membrane. Recently, Philippaert et al. suggested that the molecular mechanism through which SGs exert their therapeutic effects is by a potentiation of transient receptor potential cation channel subfamily melastatin member 5 (TRPM5) channel activity in β-cells [28]. It was strongly suggested that this monovalent cation channel was essential for the biological action of steviol and SGs, from the finding that steviol and SGs potentiate glucose-induced Ca^2+^ oscillations and thereby insulin release in WT pancreatic islets expressing TRPM5. Testing the effect of SGs on GSIS, led to the observations of Stevioside only being able to potentiate GSIS in WT islets, but not in *Trpm5^−/−^* islets. Similarly, intraperitoneal injection of 2.5 g/kg glucose plus 200 mg/kg Stevioside in fasted anaesthetized mice resulted in a significantly higher plasma insulin level 30 min post injection of Stevioside in WT mice, whereas the same effect was absent in *Trpm5^−/−^* mice. Acute oral administration of 0.5 mg/kg/day 2 h before a glucose tolerance test (GTT), also resulted in blood glucose values being significantly reduced after Stevioside treatment in WT mice, whereas no similar effect was evident from *Trpm5^−/−^* mice. The same effects were observed in alloxan-diabetic WT-recipient mice receiving WT islets, but not in WT-recipient mice receiving *Trpm5^−/−^* islets. In addition, Stevioside was reported to possess a glucose intolerance preventive effect in WT mice receiving a high-fat diet plus Stevioside compared to a high-fat diet, solely (control). Again, this effect was absent in *Trpm5^−/−^* mice, where no difference between control and the Stevioside-treated group was seen. However, expression of TRPM5 in human β-cells is relatively low, and almost absent, why further studies need to be conducted to elucidate the mechanism underlying the effects of SGs in humans [51]. 

Intravenous administration of Stevioside has been shown to induce a blood pressure reduction in hypertensive rats [45] as well as an increased water, sodium, and potassium excretion [46], suggesting a vasodilating effect of Stevioside on the kidney, eventually leading to a reduction in blood pressure. In contrast to a relatively high degree of evidence supporting the hypoglycemic and insulinotropic effects of SGs and the mechanism thereof, evidence for the exact mechanism underlying the hypotensive effect of SGs is lacking. However, it has been reported that the antihypertensive mechanism of Stevioside depends on the inhibition of Ca^2+^-influx from extracellular fluid [52]. 

The purpose for this systematic review was to evaluate the existing evidence on the effectiveness of SGs, in particular Stevioside and Reb A, on diabetic biomarkers including fasting blood glucose and insulin, lipids (cholesterol and TAGs), BP, body weight, and BMI. 

## 2. Materials and Methods 

### 2.1. Search Strategy

Electronic searches were performed in the databases PubMed and EMBASE in the time period of August to October 2018 using search terms such as Stevia, Stevioside, steviol glycoside, insulin release, antihyperglycemic agent, fasting blood glucose, triglycerides, HDL, LDL, cholesterol, BMI, systolic blood pressure, diastolic blood pressure, and glycated hemoglobin. In PubMed, the search terms were searched as MeSH terms. All synonyms and other common used terms for interventions and outcomes were included in the search. In order to be included in this review, it was required that the study design of the studies was double-blinded, randomized, controlled trials (RCTs). For inclusion purposes, it was required that the RCTs investigated the effectiveness of orally administered SG in human participants ≥18 years. In addition, the RCTs had to include a control group receiving placebo matching the intervention. Studies on SGs combined with other dietary supplements were excluded. All studies meeting the specified inclusion criteria were included in this review, without any restrictions regarding age, duration, or lifestyle adjustments. However, articles presented in languages other than English were not included. No criteria for study participants were made, thus trials in which both type 1 and 2 diabetic, hypertensive, and healthy subjects participated, were included. The outcomes of interest for this systematic review and meta-analysis were markers of type 2 diabetes, including fasting blood glucose, systolic blood pressure, diastolic blood pressure, total cholesterol, LDL, HDL, glycated hemoglobin (HbA1c), triglycerides, and BMI. 

Since it has been reported that the various SGs share the same metabolic pathway and fate [37,38], SGs are also expected to produce similar physiological effects. Thus, no inclusion criteria for a specific SG was set. 

### 2.2. Risk of Bias Assessment

The risk of bias was assessed using the Cochrane Collaboration’s tool. Study data concerning risk of bias was extracted and included in characteristics of the studies in RevMan 5.3. Each study was evaluated for unclear, low, or high risk of bias in the following criteria: random sequence generation, allocation concealment, blinding of participants and personnel, blinding of outcome assessment, incomplete outcome data, selective reporting, and other possible bias [53].

### 2.3. Data Extraction

Studies obtained from the electronic searches were considered for inclusion on the basis of title and abstract and by in-depth review of the full-text articles. Seventeen articles were selected for assessment of eligibility by two reviewers (S.R. and C.C.B.A.) for a final decision. Due to the unpublished data of Δmean and the associated SD, mean ± SD data of post-treatment for intervention and placebo groups from each included study was extracted according to number of participants and intervention effect for each outcome of interest. Baseline data between groups was checked for significant differences. Data from post-treatment was excluded in case of significant differences between group preintervention. If specified in mM, fasting blood glucose, total cholesterol, HDL, LDL, and TAG data was converted to mg/dL [54].

### 2.4. Statistical Analysis

Mean ± SD data of post-treatment for intervention and placebo groups was pooled in RevMan 5.3 [55] to compare changes after intervention between intervention and placebo groups by inverse variance and random effects model. Mean differences and 95% CI across studies were obtained by producing forest plots. In studies presenting data as mean ± SEM, SDs were derived from the reported SEMs by use of the RevMan calculator tool. Heterogeneity was investigated using *I*^2^ statistic; values of 25%, 50%, and 75% indicated low, medium, and high statistical heterogeneity, respectively. *I*^2^ > 50% indicated significant heterogeneity. 

For a proper investigation of the effect of Stevioside on diabetic biomarkers, subgroups were produced, in which studies including diabetic subjects and studies including nondiabetic subjects were analyzed independently of each other. If data was adequate, further categorial subgroup and sensitivity analyses were performed.

## 3. Results

**Figure 2:**
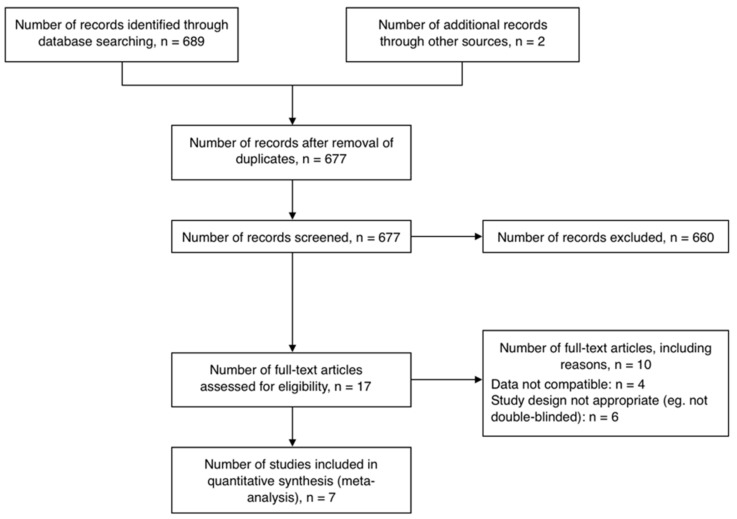
Flow chart for the number of studies screened, assessed for eligibility, and included in meta-analysis examining the effects of SGs on human health.

Six hundred and seventy-seven nonduplicate studies were obtained from electronic searches, out of which 17 eligible studies were identified (Figure 2). A total of 10 studies were excluded due to incompatible data (*n* = 4 [14,21,22,41]) and inappropriate study design (*n* = 6 [25,42,44,56,57,58]). Seven studies comprising nine RCTs and a total of 462 participants were included in this review [15,16,17,18,19,43,59]. All studies were of parallel design except one [18], and placebo-controlled by administration of placebo matching the intervention (Table 1). Three RCTs included participants with hypertension [16,19,59], two of which included subjects with mild hypertension [19,60], four RCTs included diabetic participants [15,18,43], of which one included type 1 diabetic subjects [15], one RCT included untreated hyperlipidemic patients [17], while only one RCT included healthy subjects [15]. The SG Stevioside was used as intervention for all studies at a daily dose varying from 200–1500 mg. Variations were observed in number of times the supplements (both intervention and placebo) were administered per day. The duration of Stevioside or placebo administration ranged from 4 h to 2 years.

**Figure 3:**
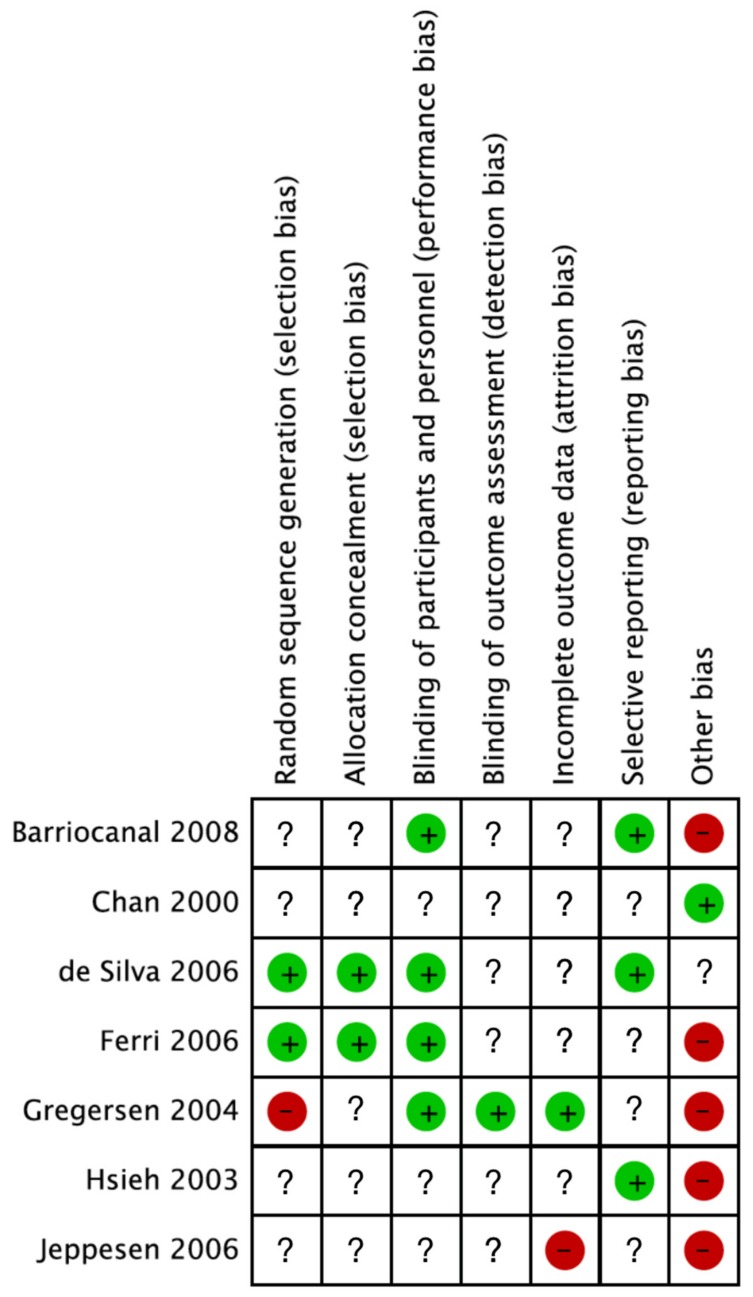
Risk of bias summary of studies included in meta-analysis examining the effect of SGs on human health. Adequate reporting is marked by either green (no bias) or red (bias). Nonadequate information about the bias in question is marked by “?”.

The contents of placebos varied between studies and were specified in only three studies, including maize starch [18,43] and talcum [17]. For the rest of the included studies, “matching placebo” was the only annotation regarding contents of placebos. Additionally, lifestyle adjustments also varied across the studies, and the majority of included studies did not specify their sources of funding. Discrepancies between studies were observed by assessing the risk of bias (Figure 3). Two studies adequately reported randomization and allocation concealment procedures, and four studies adequately reported blinding of participants and personnel, while only one study adequately reported blinding of outcome assessors. Other biases, such as continuation of medicine, were detected in several of the studies. However, the majority of included studies lacked information regarding the assessed biases.

### 3.1. Effect of Steviol Glycosides on BMI

**Figure 4:**
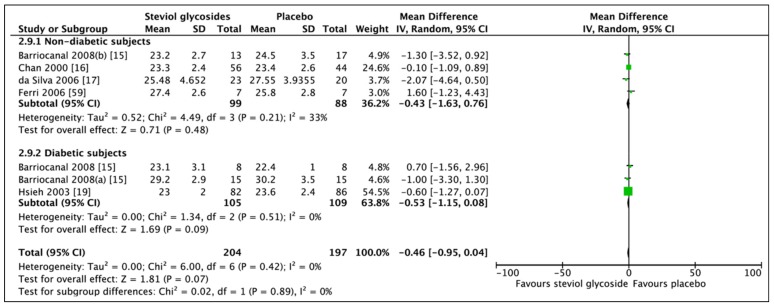
Effect of SGs on BMI (kg/m^2^) CI: confidence interval; MD: mean difference; RCTs: randomized controlled trials; SD: standard deviation.

A total of five studies (seven RCTs, *n* = 401) were included in the meta-analysis of BMI (Figure 4). No overall effect on BMI was found between administration of SGs and placebo (MD: −0.46, CI: −0.95–0.04). For subgroup analysis, the meta-analysis of BMI included four studies (four RCTs, *n* = 187) in the nondiabetic group and two studies (three RCTs, *n* = 214) in the diabetic group. In nondiabetic subjects, BMI was not affected by the administration of SGs for 90 days–1 year (MD: −0.43, 95% CI: −1.63–0.76, *p* = 0.48). Meta-analysis of the diabetes subgroup also revealed a non-significant effect of SG administration for 3 months–2 years. A trend towards a lower BMI was observed (MD: −0.53, CI: −1.15–0.08, *p* = 0.09); however, this did not reach statistical significance. Some heterogeneity was reported to be present in the nondiabetic subgroup (*I*^2^ = 33%, *p* = 0.21). No significant heterogeneity was found in neither nondiabetic or diabetes subgroups (*I*^2^ = 0%, *p* = 0.51). Sensitivity analysis showed two studies significantly contributing to the heterogeneity of the nondiabetic group [17,60].

### 3.2. Effect of Steviol Glycosides on Blood Pressure

**Figure 5:**
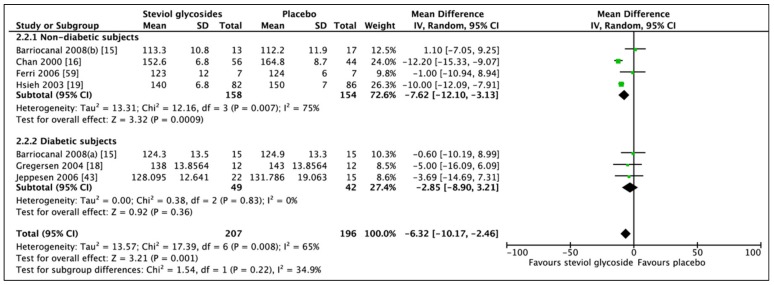
Effect of SGs on systolic blood pressure (SBP, mmHg) CI: confidence interval; MD: mean difference; RCTs: randomized controlled trials; SD: standard deviation.

Six studies (seven RCTs, *n* = 403) reported adequate and compatible data for statistical analyses (Figure 5). Meta-analysis of these RCTs showed a significant difference in SBP when comparing SGs and placebo (MD: −6.32 mm Hg, CI: −10.17 to −2.46, *p* = 0.001). However, heterogeneity was reported, although not significant (*I*^2^ = 34.9%, *p* = 0.22). Subgroup analysis of four RCTs and three RCTs for the nondiabetic and diabetic group, respectively, revealed only a significant change in SBP in the nondiabetic subgroup between SGs and placebo (MD: −7.62 mm Hg, CI: −12.10, −3.13, *p* = 0.0009). No significant difference was observed in the diabetic subgroup (MD: −2.85 mm Hg, CI: −8.90 to 3.21, *p* = 0.36). Significant heterogeneity was reported in the nondiabetic subgroup (I^2^ = 75%, *p* = 0.007), while no heterogeneity was evident from the diabetic subgroup (*I*^2^ = 0%, *p* = 0.83). Sensitivity analyses revealed differences in outcome when comparing only studies including hypertensive subjects. In fact, further subgroup analysis of the effect of SGs on SBP in nondiabetic hypertensive subjects (two RCTs, *n* = 268), revealed a significant reduction in systolic blood pressure (MD: −10.78 mm Hg, CI: −12.84 to −8.72, *p* < 0.00001) with non-significant heterogeneity between the studies (*I*^2^ = 24%, *p* = 0.25). 

**Figure 6:**
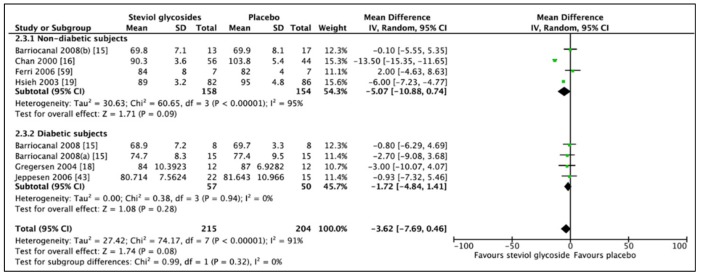
Effect of SGs on diastolic blood pressure (DBP, mmHg) CI: confidence interval; MD: mean difference; RCTs: randomized controlled trials; SD: standard deviation.

An additional RCT was included in the meta-analysis of DBP, which was excluded in the analysis of SBP due to significant baseline differences between groups. Thus, six studies (eight RCTs, n = 494) were included in the meta-analysis concerning the effects of SGs on DBP (Figure 6). No overall effect was found when combining all studies (MD: −3.62 mm Hg, CI: −7.69 to 0.49, *p* = 0.08) and significant heterogeneity was found (*I*^2^ = 91%, *p* < 0.00001). Subgroup analyses showed non-significant differences for both nondiabetic (MD: −5.07 mm Hg, CI: −10.88 to 0.74, *p* = 0.09) and diabetic subgroups (MD: −1.72 mm Hg, CI: −4.84 to 1.41, *p* = 0.28). No heterogeneity was found between studies included in the diabetic subgroup analysis, while significant heterogeneity was reported between studies included in the nondiabetic subgroup analysis (*I*^2^ = 95%, *p* < 0.00001). Comparing only studies including nondiabetic, hypertensive subjects revealed the same findings as to when comparing nondiabetic subjects regardless of blood pressure.

### 3.3. Effect of Steviol Glycosides on Fasting Blood Glucose

**Figure 7:**
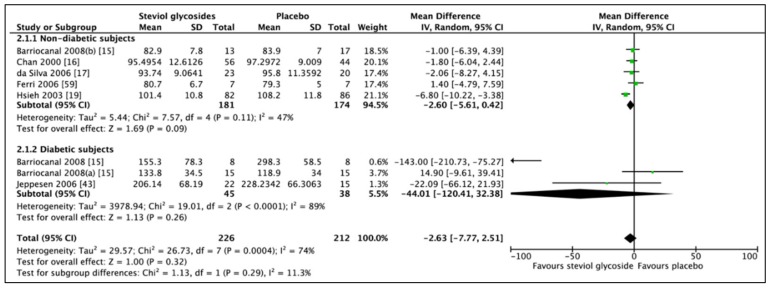
Effect of SGs on fasting blood glucose (FBG, mg/dL) CI: confidence interval; MD: mean difference; RCTs: randomized controlled trials; SD: standard deviation.

Meta-analysis of six studies (8 RCTs, *n* = 438) showed no significant difference in FBG in favour of SG (Figure 7; MD: −2.63 mg/dL, CI: −7.77 to 2.51, *p* = 0.32). Significant heterogeneity was found between the included studies (*I*^2^ = 74%, *p* = 0.0004). Likewise, when analyzing on the basis of subgroups, diabetic status was shown not to affect the direction of the results (nondiabetic subgroup: MD: −2.60 mg/dL, CI: −5.61 to 0.42, *p* = 0.09 and diabetic subgroup: MD: −44.01, CI: −120.41 to 32.38, *p* = 0.26). Significant heterogeneity was reported for both groups (*I*^2^ = 47%, *p* = 0.11 and *I*^2^ = 89%, *p* < 0.0001, respectively). One RCT included type 1 diabetic subjects. Performing a sensitivity analysis, leaving these data out, resulted in a decreased heterogeneity. Furthermore, by conducting a subgroup analysis including studies prohibiting any type of medications (four RCTs, *n* = 216), including antihypertensive and antidiabetic medications, no significant difference in FBG was observed (MD: −1.19, CI: −4.23 to 1.85, *p* = 0.44). However, no heterogeneity was observed between included studies (*I*^2^ = 0%, *p* = 0.64). 

### 3.4. Effect of Steviol Glycosides on Cholesterol

**Figure 8:**
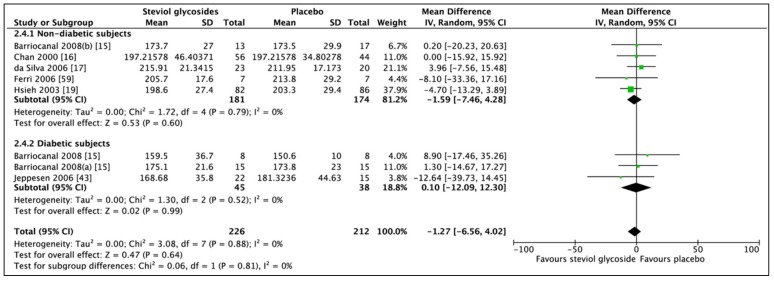
Effect of SGs on total cholesterol (mg/dL) CI: confidence interval; MD: mean difference; RCTs: randomized controlled trials; SD: standard deviation.

A non-significant difference in total cholesterol between SG and placebo (Figure 8; MD: −1.27 mg/dL, CI: −6.56 to 4.02, *p* = 0.64) was revealed by a meta-analysis of six studies (eight RCTs, *n* = 438). Analysis of five RCTs (*n* = 355) including nondiabetic subjects (MD: −1.59 mg/dL, CI: −7.46 to 4.28, *p* = 0.60) and 3 RCTs (*n* = 83) including diabetic subjects (MD: 0.10 mg/dL, CI: −12.09 to 12.30, *p* = 0.99) did not change the direction of meta-analytic results. No heterogeneity was found between studies included in the subgroup analysis of nondiabetic subjects (*I*^2^ = 0%, *p* = 0.79). The same was evident from the subgroup analysis of diabetic subjects (*I*^2^ = 0%, *p* = 0.52). 

**Figure 9:**
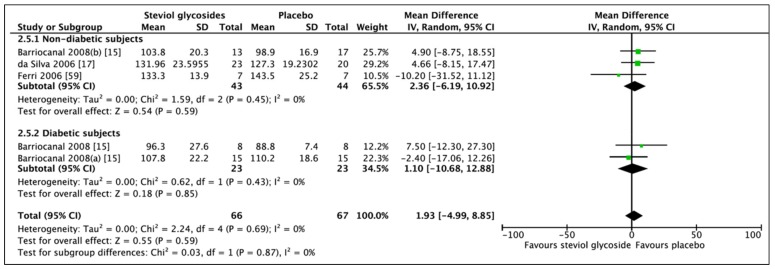
Effect of SGs on low-density lipoprotein cholesterol (LDL-C, mg/dL) CI: confidence interval; MD: mean difference; RCTs: randomized controlled trials; SD: standard deviation.

**Figure 10:**
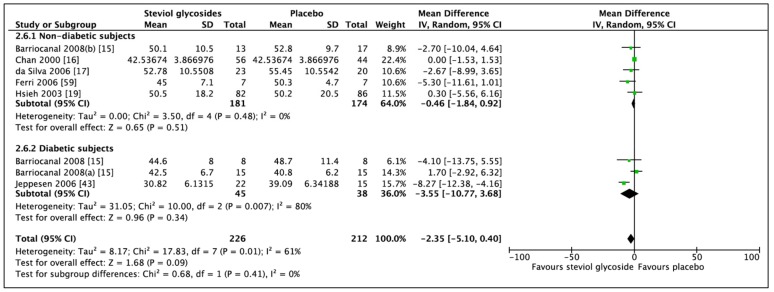
Effect of SGs on high-density lipoprotein cholesterol (HDL-C, mg/dL) CI: confidence interval; MD: mean difference; RCTs: randomized controlled trials; SD: standard deviation.

Similar findings were observed for LDL-C and HDL-C ( Figure 9; Figure 10, respectively).

However, while no heterogeneity was observed between studies included in the meta-analysis of LDL-C, heterogeneity was reported between studies included in the diabetic subgroup analysis of HDL-C (Figure 10). 

Sensitivity analysis revealed that the study by Barriocanal et al. (RCT including type 2 diabetics) significantly contributed to the heterogeneity of the diabetic subgroup. By removing the data from the analysis, the HDL-C was found to be significantly decreased in favour of SG (MD: −7.63 mg/dL, CI: −11.41 to −3.85, *p* < 0.0001).

**Figure 11:**
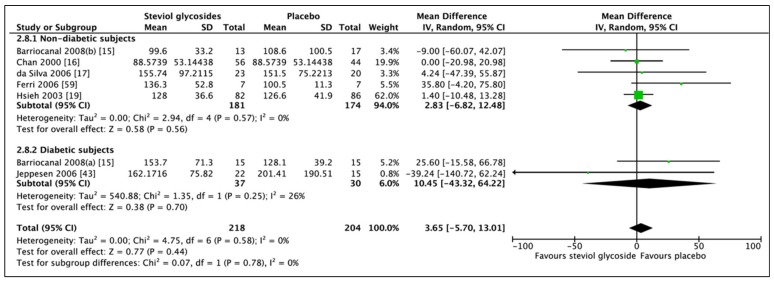
Effect of SGs on triglycerides (TAGs, mg/dL) CI: confidence interval; MD: mean difference; RCTs: randomized controlled trials; SD: standard deviation.

Meta-analysis of six studies (seven RCTs, *n* = 422) showed an overall non-significant difference in TAGs between SGs and placebo (Figure 11; MD: 3.65 mg/dL, CI: −5.70 to 13.01, *p* = 0.44). Subgroup analyses did not change the direction of results in neither nondiabetic (MD: 2.83 mg/dL, CI: −6.82 to 12.48, *p* = 0.58) or diabetic subgroups (MD: 10.45 mg/dL, CI: −43.32 to 64.22, *p* = 0.70). No heterogeneity was found between studies in the nondiabetic subgroup (*I*^2^ = 0%, *p* = 0.57). However, heterogeneity was found for the diabetic subgroup (*I*^2^ = 26%, *p* = 0.25). 

### 3.5. Effect of Steviol Glycosides on Glycated Hemoglobin

**Figure 12:**
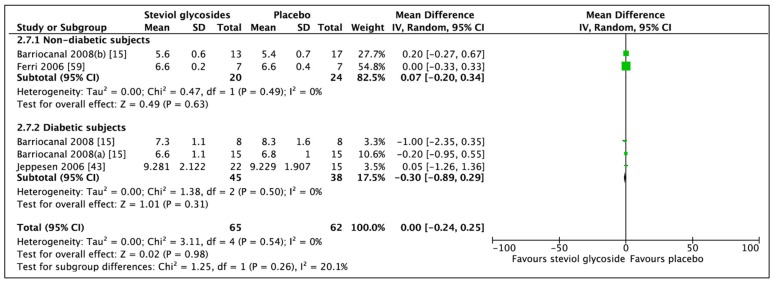
Effect of SGs on glycated hemoglobin (HbA1c, %) CI: confidence interval; MD: mean difference; RCTs: randomized controlled trials; SD: standard deviation.

Three studies (five RCTs, *n* = 127) reported adequate data for statistical analysis. Meta-analysis of these RCTs showed a non-significant difference in HbA1c between SGs and placebo (Figure 12; MD: 0.00%, CI: −0.24 to 0.25, *p* = 0.98). Subgroup analyses did not change the direction of the outcome (nondiabetic subjects: MD: 0.07%, CI: −0.20 to 0.34, *p* = 0.63 and diabetic subjects: MD: −0.30%, CI: −0.89 to 0.29, *p* = 0.31). No heterogeneity was found between studies included in the overall analysis (*I*^2^ = 0%, *p* = 0.54) or in the subgroup analyses of nondiabetic (*I*^2^ = 0%, *p* = 0.49) or diabetic subjects (*I*^2^ = 0%, *p* = 0.50).

## 4. Discussion

This systematic review and meta-analysis suggest that administration of SGs causes non-significant reductions in BMI, diastolic blood pressure, fasting blood glucose, total cholesterol, and HDL-C in nondiabetic subjects. Even though the data did not reach statistical significance, it is worth mentioning that SG administration tended to reduce the diastolic blood pressure (*p* = 0.09). A non-significant increase in TAGs in favour of placebo was also observed in non-diabetic subjects. Furthermore, a significant decrease in systolic blood pressure in favour of SGs was found in both overall and subgroup analysis of non-diabetic subjects, although the heterogeneity was found to be significant. However, when exclusively comparing data from long-term studies including non-diabetic, hypertensive subjects, a significant reduction with no heterogeneity between studies was observed. Only two long-term studies included non-diabetic, hypertensive subjects.

In the diabetic subgroup, a non-significant increase in favour of placebo in LDL-C was suggested. Also, non-significant reductions in BMI in favour of SGs was observed. However, this decrease tended to reach statistical significance (*p* = 0.09).

Non-significant reductions in favour of SGs of systolic blood pressure, diastolic blood pressure, fasting blood glucose, HbA1c, and HDL-C were suggested in diabetic subjects. Furthermore, non-significant increases in favour of placebo of total cholesterol and LDL were evident from this meta-analysis. Our results also showed an increasing effect of SGs on LDL-C and TAGs. 

Our findings partially corroborate the findings from a previous systematic review and meta-analysis, suggesting beneficial effects of Stevioside on cardiovascular risk factors [24]. Similarities in the included articles exist between the systematic review and meta-analysis by Onakpoya et al. and the current study. However, two papers investigating the effect of Reb A on several outcomes of interest included in the study by Onakpoya et al. were excluded from this study due to incompatible data. Thus, only studies using Stevioside as intervention were included. Interestingly, Onakpoya et al. reported a non-significant effect of Reb A on outcomes such as SBP, DBP, fasting blood glucose, and lipid profile. Also, one additional trial not available from the previous systematic review and meta-analysis was included in the current study. In addition, this study further developed this area of investigation by another approach. We aimed to investigate the effect of SGs on human health, particularly diabetes biomarkers, including BMI, SBP, DBP, FBG, total cholesterol, LDL-C, HDL-C, TAGs, and HbA1c. 

Similar to the observations by Onakpoya et al., significant heterogeneity was found in some of the overall analyses in the current meta-analysis. Thus, the results from this systematic review and meta-analysis should be interpreted with caution. Substantial variations were observed in trial design, variation in daily doses of Stevioside, duration of intervention, and differences in protocols regarding lifestyle, including continuation of antihypertensive and antidiabetic medications across included studies. However, some of the subgroup analyses showed small or no heterogeneity, indicating true directions of the results of this intervention. 

Since the effects of SGs have been investigated on a variety of outcomes in both humans and animals, and it has been reported that SGs exhibit both hypotensive, hypoglycemic, and hypolipidemic actions, the purpose for this study was to gather data from clinical trials and compare these. 

From clinical trials, it has been suggested that administration of Stevia and SGs do not exhibit any body weight or BMI-lowering effect compared with placebo treatment, consistent with this meta-analysis [16,17,19,22]. Additionally, no effects of Stevia affecting satiety and energy intake to a significant extent have been reported [14,25], suggesting the non-caloric profile of SGs being responsible for the possible reduction in body weight or BMI if presented [48]. Most of the included studies incorporated lifestyle adjustments regarding diet and physical activity into their study procedure, which might have affected the results since these factors play a crucial role in affecting weight and BMI profiles of the subjects. 

From a previous systematic review, Stevia intake was found to produce an increase in blood pressure when combining findings from studies performed for 1–3 months. In contrast, a lowering of blood pressure in hypertensive patients consuming Stevia for 1–2 years was reported [59]. In a 2-year study, consumption of 1500 mg/day Stevioside was found to contribute to healthy blood pressure regulation by inducing vasorelaxation [61]. Further antihypertensive effects of Stevia have been reported by Tomas et al., showing a systolic and diastolic blood pressure decreasing effect in mild-hypertensive subjects by administration of 750 mg Stevia [62]. Asides from the reported effect sizes for the reduction in blood pressures appear small, even small reductions may be beneficial in the prevention and management of hypertension. 

Even though hypoglycemic effects and the underlying mechanisms of SGs are well established to some extent from several animal studies as already reported [26,27,28,29,31,32], no indications of reductions of blood glucose levels or in HbA1c in favour of SGs was found from the current study. No reductions in blood glucose levels or HbA1c were expected for non-diabetics from findings showing a stimulation in insulin release only at high glucose concentrations [26,27]. Thus, it is suggested that Stevioside elicits its beneficial effect by stimulating insulin release only in the diabetic state. Unexpectedly, no significant reductions of blood glucose levels in favour of SGs were found from subgroup analysis of diabetic subjects. However, due to the inconsistency between protocols across the included studies, some diabetic subjects were allowed to continue their antidiabetic medications, while other prohibited this medication before initiation of intervention, which might have affected the results to a great extent. 

The same is applicable for the results of the lipid profile. Administration of a range of oral antihyperglycemic medications have been shown to affect the lipid profile in type 2 diabetics [63]. In previous studies, Stevioside alone or in combination with soy-protein isolate has been shown to decrease plasma lipids in the type 2 diabetic GK rat [64] and in high-fat diet fed mice [65]. Sharma et al. studied the effect of consumption of Stevia extract on 20 selected hypercholesterolemic women, and found a significant reduction in cholesterol, TAGs, and LDL-C, and a significant increase in HDL-C [66]. Only one of the included studies dealt with hyperlipidemic subjects, possibly explaining the obtained results. Significant differences were not found from the systematic review and meta-analysis by Onakpoya et al., suggesting that the results from animal studies do not translate to noticeable changes in humans. The duration of the included studies might have affected the results, hence why studies with a longer duration are of interest. Findings from animal studies have shown Stevia supplements to reduce TAGs [67], while this is not found from the current study. Actually, the contrary was observed from the overall analysis and the subgroup analysis of non-diabetic subjects. Unfortunately, only two studies concerning type 2 diabetics were included, meaning no statistically appropriate outcome was achieved. 

Due to inconsistencies between study protocols regarding regulations in lifestyle such as diet and physical activity, the majority of these findings should be interpreted with caution, since lifestyle changes might improve the cardiovascular risk profile [68,69]. It is uncertain whether these lifestyle adjustments exclusively led to the observed small changes in some of the outcomes or enhanced or blunted the effects of SGs. 

It would have been preferable to perform additional subgroup analyses to further elucidate the true effects of SGs on the outcomes. However, due to the limited quantity of included studies and the resultant limited number of participants, this was assessed not to be sufficient. To be able to perform true and statistically comparable subgroup analyses, it was determined beforehand that three or more studies presenting adequate data for statistical analysis should be included. For some of the outcomes, this was not the case, hence no conclusions should be made from these subgroup analyses. To be able to achieve as many subgroup analyses as possible, one RCT including T1D patients was included in the subgroup analyses of diabetic subjects. Due to major differences in etiology and pathogenesis between T1D and T2D [70], bias might have occurred. In addition, for future aspects, ∆mean and the associated SDs should be extracted from the articles rather than comparing placebo and intervention post-treatment data. The fact that the current meta-analysis was conducted on the basis of the post-treatment data might have resulted in exclusion of important data. 

Onakpoya et al. requested information and missing data from trial investigators of included studies, and imputed SDs [71] from reported *p* values or upper limits for the significance levels, assuming that the SDs for each outcome reported were the same in both intervention and comparator groups, which might have resulted in bias [24]. To further improve this systematic review and meta-analysis, missing data should be requested from trial investigators.

Lastly, it is noteworthy that the majority of the clinical trials included in the current study did not aim to investigate the effects of SGs on the outcomes of interest. Rather, the efficacy and tolerability of SGs were examined to be able to confirm or deny the possible harmful effects of SGs in hypertensive or diabetic subjects.

Further clinical trials, in particular RCTs, investigating the effects of Stevioside are required. The trials should investigate the effects of diabetic biomarkers and include diabetic subjects, last for more than 6 months, and prohibit antidiabetic medications if possible. Furthermore, the diabetic subjects included in the trials should be newly diagnosed to avoid limited beta cell function of the patients. Studies should include descriptions of the placebo interventions [72].

## 5. Conclusions

From this systematic review and meta-analysis including data from published RCTs, Stevioside is suggested to generate reductions in blood pressure, in particular in SBP. Differences in favour of SGs of other outcomes were also evident, but not to a significant extent. Since the majority of included studies are based on safety of SG as a sweetener rather than a pharmaceutical treatment, the doses used are probably not high enough to induce any physiological changes. 

In addition, continuation of antidiabetic and antihypertensive medications might have affected the final outcomes of the individual studies and thereby the overall outcome of the current study. Further clinical trials including diabetic subjects are warranted.

## Figures and Tables

**Table 1:** Characteristics of included randomized controlled trials (RCTs).

Study ID	Outcomes	Description of Participants	Study Duration	Intervention	Placebo	Lifestyle Adjustments	Funding
**Barriocanal 2008 ^a^ [** 15 **]**	BMI, SBP, DBP, FBG, TC, HDL-C, LDL-C, TAG, HbA1c	Men and women with or without T1D or T2D (n = 86)	3 months	Stevioside 750 mg/day Purity: ≥92%	Matching placebo (content not specified)	Continuation of antihypertensive and antidiabetic medication during intervention	Not specified
**Chan 2000 [** 16 **]**	BMI, SBP, DBP, FBG, TC, HDL-C, TAG	Mild to moderate hypertensive men and women (n = 100)	1 year	Stevioside 750 mg/day Purity: Not available	Matching placebo (content not specified)	Prohibition of antihypertensive medication. Dietary counselling	Not specified
**da Silva 2006 [** 17 **]**	BMI, FBG, TC, HDL-C, LDL-C, TAG	Untreated hyperlipidemic men and women (n = 43)	90 days	Stevioside 200 mg/day Purity: Not available	Talcum	Normal lifestyle	Public institutions
**Ferri 2006 [** 60 **]**	BMI, SBP, DBP, FBG, TC, HDL-C, LDL-C, TAG, HbA1c	Untreated mild-hypertensive men and women (n = 14)	24 weeks	Stevioside: 7 weeks: 3.75 mg/kg/day 11 weeks: 7.0 mg/kg/day 6 weeks: 15.0 mg/kg/day Purity: Not available	Talcum	Normal lifestyle	Government
**Gregersen 2004 ^b^ [** 18 **]**	SBP, DBP	Overweight and obese men and women with T2D (n = 12)	4 h	Stevioside 1000 mg Purity: 91%	Maize starch	Prohibition of oral antidiabetic medication prior to intervention	Public institutions
**Hsieh 2003 [** 19 **]**	BMI, SBP, DBP, FBG, TC, HDL-C, TAG	Mild-hypertensive men and women (n = 168)	2 years	Stevioside 1500 mg/day Purity: Not available	Matching placebo (content not specified)	Continuation of antihypertensive medication during intervention	Not specified
**Jeppesen 2006 [** 43 **]**	SBP, DBP, FBG, TC, HDL-C, TAG, HbA1c	T2D men and women (n = 37)	3 months	Stevioside 1500 mg/day Purity: Not available	Maize starch	Prohibition of antidiabetic medication two weeks prior to intervention	Not specified

BMI: body mass index; SBP: systolic blood pressure; DBP: diastolic blood pressure; FBG: fasting blood glucose; TC: total cholesterol; HDL-C: high-density lipoprotein cholesterol; LDL-C: low-density lipoprotein cholesterol; TAG: triglyceride; HbA1c: glycated hemoglobin; T1D: type 1 diabetes; T2D: type 2 diabetes. **^a^** Three RCTs in one study. Group 1: T1D; Group 2: T2D; Group 3: Healthy. **^b^** Cross-over trial.
